# Supplementary material for: Adaptive evolution of nontransitive fitness in yeast
Source: eLife. 2020 Dec 29;9:e62238. doi: 10.7554/eLife.62238 (PMC7886323; doi:10.7554/eLife.62238)
Supplement: Supplementary file 1. — A caret (∧) indicates that a population is heteroplasmic for variants listed. An asterisk (*) indicates that the mutation results in loss of PCR primer binding sites thereby preventing further characterization. [file elife-62238-supp1.docx]

| **Change Killer Phenotype** |  |  |  |
| --- | --- | --- | --- |
| **Population** | **Killing Ability** | **Immunity** | **K1 Mutations (subunit)** |
| BYS1-D04 | 0.00 | 0.00 | D106G (α), Y185N (γ) |
| BYS1-E05 | 0.00 | 0.00 | I243I (β), D253N (β) |
| BYS1-E07 | 0.00 | 0.00 | M1, LA not detected |
| BYS2-A06 | 0.00 | 0.00 | G289S (β) |
| BYS2-B09 | 0.00 | 0.00 | -1 bp frameshift (α) |
| BYS2-C08 | 0.00 | 0.00 | D253N (β) |
| BYS2-E10 | 0.00 | 0.00 | D106G (α), K296E (β) |
| BYS2-G10 | 0.00 | 0.00 | I292M (β) |
| BYS2-H10 | 0.00 | 0.00 | I292M (β) |
| BYS2-B04 | 0.00 | 0.33 | Y236C (β) |
| BYS1-A12 | 0.33 | 0.67 | L27P (δ), N90N (α) |
| BYS1-A02 | 0.00 | 1.00 | S13S (δ), D106G (α) |
| BYS1-A05 | 0.00 | 1.00 | I292M (β) |
| BYS1-A08 | 0.00 | 1.00 | D106G (α), H213R (γ) |
| BYS1-B02 | 0.00 | 1.00 | D106G (α) |
| BYS1-B03 | 0.00 | 1.00 | I292M (β) |
| BYS1-B06 | 0.00 | 1.00 | D168G (γ), I292M (β) |
| BYS1-C02 | 0.00 | 1.00 | I292M (β) |
| BYS1-C06 | 0.00 | 1.00 | I292M (β) |
| BYS1-C10 | 0.00 | 1.00 | S66S (α), D106G (α) |
| BYS1-D06 | 0.00 | 1.00 | D106G (α), Δ 878 bp ^ |
| BYS1-D07 | 0.00 | 1.00 | I292M (β) |
| BYS1-D08 | 0.00 | 1.00 | I292M (β), uncharacterized mutation* |
| BYS1-G07 | 0.00 | 1.00 | D106G (α) |
| BYS2-A01 | 0.00 | 1.00 | I292M (β) |
| BYS2-A04 | 0.00 | 1.00 | T36T (δ), Y236H (β), I292M (β) |
| BYS2-A11 | 0.00 | 1.00 | P47S (α), D50V (α) |
| BYS2-B11 | 0.00 | 1.00 | D106G (α) |
| BYS2-C03 | 0.00 | 1.00 | Y236H (β), I292M (β) |
| BYS2-C05 | 0.00 | 1.00 | K244R (β), V256M (β) |
| BYS2-C09 | 0.00 | 1.00 | T195T (γ), I292M (β) |
| BYS2-E02 | 0.00 | 1.00 | D106G (α) |
| BYS2-E04 | 0.00 | 1.00 | K283K (β), I292M (β) |
| BYS2-E06 | 0.00 | 1.00 | D106G (α), F222L (γ) |
| BYS2-E07 | 0.00 | 1.00 | D106G (α) |
| BYS2-E11 | 0.00 | 1.00 | I292M (β), Δ1177 bp ^ |
| BYS1-B04 | 0.33 | 1.00 | D253N (β) |
| BYS1-B08 | 0.33 | 1.00 | R149H (γ) |
| BYS1-B10 | 0.33 | 1.00 | Y282C (β) |
| BYS1-D10 | 0.33 | 1.00 | W277R (β) |
| BYS1-E08 | 0.33 | 1.00 | Q184R (γ), N307D (β), D308N (β) |
| BYS2-A03 | 0.33 | 1.00 | D106G (α) |
| BYS2-F05 | 0.33 | 1.00 | D253N (β) |
| BYS2-G05 | 0.33 | 1.00 | Y219H (γ) |
| BYS1-A03 | 0.67 | 1.00 | D253N (β) |
| BYS1-A09 | 0.67 | 1.00 | N280D (β) |
| BYS1-C05 | 0.67 | 1.00 | D253N (β) |
| BYS1-D03 | 0.67 | 1.00 | D253N (β) |
| BYS1-E02 | 0.67 | 1.00 | T223I (γ) |
| BYS1-H07 | 0.67 | 1.00 | W291R (β) |
| BYS1-H11 | 0.67 | 1.00 | D253N (β) |
| BYS2-A10 | 0.67 | 1.00 | T64T (α), D253N (β) |
| BYS2-B08 | 0.67 | 1.00 | D253N (β) |
| BYS2-C01 | 0.67 | 1.00 | D253N (β) |
| BYS2-C12 | 0.67 | 1.00 | D253N (β) |
| BYS2-D02 | 0.67 | 1.00 | D253N (β) |
| BYS2-F02 | 0.67 | 1.00 | H214H (γ), D253N (β) |
|  |  |  |  |
| **Retain Killer Phenotype** |  |  |  |
| **Population** | **Killing Ability** | **Immunity** | **K1 Mutations (subunit)** |
| BYS1-A04 | 1.00 | 1.00 |  |
| BYS1-A07 | 1.00 | 1.00 |  |
| BYS1-F05 | 1.00 | 1.00 |  |
| BYS1-G01 | 1.00 | 1.00 |  |
| BYS1-G02 | 1.00 | 1.00 |  |
| BYS2-C06 | 1.00 | 1.00 | L269L (β) |
| BYS2-D06 | 1.00 | 1.00 |  |
| BYS2-D07 | 1.00 | 1.00 |  |
| BYS2-E01 | 1.00 | 1.00 |  |
| BYS2-E03 | 1.00 | 1.00 |  |
