# Supplementary material for: Adaptive evolution of nontransitive fitness in yeast
Source: eLife. 2020 Dec 29;9:e62238. doi: 10.7554/eLife.62238 (PMC7886323; doi:10.7554/eLife.62238)
Supplement: Supplementary file 2. [file elife-62238-supp2.docx]

| **Nucleotide Substitution** | **K1**  (this study) | **K1**  (*Greig 2008*) | **Gag-Pol LA** (*Diamond 1989, Suzuki 2015*) | **Nuclear**  (*Lang 2013, Fisher 2018, Marad 2018*) | **Nuclear – Common Targets**  (*Lang 2013, Fisher*  *2018, Marad 2018*) |
| --- | --- | --- | --- | --- | --- |
| C:G>U:A (Ts) | 10 (27%) | 17 (33%) | 17 (35%) | 1174 (31%) | 47 (23%) |
| A:U>G:C (Ts) | 22 (59%) | 24 (47%) | 19 (40%) | 558 (15%) | 24 (12%) |
| A:U>C:G (Tv) | 1 (3%) | 5 (10%) | 1 (2%) | 348 (9%) | 11 (5%) |
| A:U>U:A (Tv) | 4 (11%) | 0 (0%) | 6 (13%) | 368 (10%) | 20 (10%) |
| C:G>A:U (Tv) | 0 (0%) | 4 (8%) | 4 (8%) | 850 (22%) | 65 (32%) |
| C:G>G:C (Tv) | 0 (0%) | 1 (2%) | 1 (2%) | 530 (14%) | 35 (17%) |
| Transitions | 32 | 41 | 36 | 1732 | 71 |
| Transversions | 5 | 10 | 12 | 2096 | 131 |
| R | 6.4 | 4.1 | 3.0 | 0.8 | 0.5 |
